# PDGFRß targeted positron emission tomography as a non-invasive biomarker for activated hepatic stellate cells: lasts steps before clinical translation

**DOI:** 10.1186/s41181-025-00410-2

**Published:** 2025-12-13

**Authors:** Chittampalli N. Yashaswini, Bogdan Mitran, Natalia Papadopoulos, Olivia Wegrzyniak, John Löfblom, Helena Nordström, Irina Velikyan, Ayman Abouzayed, Lars Johansson, Per Hagmar, Michael Wagner, Fredrik Y. Frejd, Olle Korsgren, Carl-Henrik Heldin, Scott L. Friedman, Olof Eriksson

**Affiliations:** 1 Division of Liver Diseases, Icahn School of Medicine at Mount Sinai, 1425 Madison Ave., Room 11-70C, Box 1123, New York, NY 10029-6574 USA; 2 Science for Life Laboratory, Department of Medicinal Chemistry, Uppsala University, Dag Hammarskjölds Väg 14C, 3Tr, 751 83 Uppsala, Sweden; 3 Antaros Tracer AB, Uppsala, Sweden; 4 Department of Medical Biochemistry and Microbiology, Uppsala University, Uppsala, Sweden; 5 Department of Protein Science, Division of Protein Engineering, KTH Royal Institute of Technology, Stockholm, Sweden; 6 Science for Life Laboratory, Drug Discovery and Development Platform, Department of Chemistry-BMC, Uppsala University, Uppsala, Sweden; 7 PET Center, Uppsala University Hospital, Uppsala, Sweden; 8 Antaros Medical AB, Uppsala, Sweden; 9 Dewpoint Therapeutics GmbH, Frankfurt, Germany; 10 Department of Immunology, Genetics and Pathology, Uppsala University, Uppsala, Sweden; 11 Affibody AB, Solna, Sweden

**Keywords:** Platelet-derived growth factor receptor, Hepatic stellate cells, Fibrogenesis, Liver fibrosis, MASH, PET, Affibody molecule

## Abstract

**Background:**

Activated hepatic stellate cells (aHSCs) are the key cell population in the injured liver driving fibrogenesis. aHSCs express platelet-derived growth factor receptor beta (PDGFRß), which is absent from quiescent HSCs. PDGFRß is therefore an attractive target of PET tracers for imaging of fibrogenesis. Here, we present the pharmacological characterization of [^68^Ga]Ga-DOTA-Cys-ATH001 in preparation for clinical translation and further confirm PDGFRß as a biomarker of activated HSCs in liver disease by single cell sequencing.

**Methods:**

The expression of PDGFRß in subpopulations of HSCs was evaluated in scRNAseq datasets from both a mouse and human liver samples. DOTA-Cys-ATH001 was evaluated for affinity and mechanism of binding to PDGFRß. [^68^Ga]Ga-DOTA-Cys-ATH001 was evaluated for binding in vitro in mouse and human liver biopsies. The in vivo stability, biodistribution, pharmacokinetics, dosimetry and microdosing toxicology were evaluated in rats and pigs.

**Results:**

PDGFRß expression was specifically upregulated in activated HSCs. [^68^Ga]Ga-DOTA-Cys-ATH001 could differentiate fibrotic liver from healthy liver. The binding co-localized with tissue areas positive for collagen deposition and PDGFRß immunostaining. Based on the microdosing toxicology study the no observed adverse effect level was at least 1000 µg/kg, suggesting that the intended clinical PET scan dose is safe for use. Dosimetry calculations of [^68^Ga]Ga-DOTA-Cys-ATH001 predicted an effective dose in human amenable to repeated examinations.

**Conclusions:**

The data presented here suggests that PDGFRβ PET imaging with [^68^Ga]Ga-DOTA-Cys-ATH001 has potential for non-invasive detection of activated HSCs. Clinical translation of [^68^Ga]Ga-DOTA-Cys-ATH001 is ongoing.

**Supplementary Information:**

The online version contains supplementary material available at 10.1186/s41181-025-00410-2.

## Background

Fibrosis is the pathological accumulation of extracellular matrix (ECM) components, including collagens, in tissue. Liver fibrosis is associated with chronic liver injury, among the most common of which is metabolic dysfunction-associated steatotohepatitis (MASH), which is emerging as a global health challenge (Parola and Pinzani 2019).

The extent of fibrosis in tissue is determined by the balance between production of new collagen (fibrogenesis) and the degradation and clearance of collagen fibrils (fibrolysis). Activated hepatic stellate cells (HSCs) are the main source of collagen deposition in the liver, and thus the main cells to monitor in assessing fibrogenesis (Higashi et al. 2017; Cogliati et al. 2023). HSCs become activated by several drivers including hepatic inflammation, pressure or liver injury (Tsuchida and Friedman 2017). Reduction of fibrogenesis by targeting the drivers leading to HSC activation is a promising interventional strategy to treat liver fibrosis, but pharmaceutical development is challenging owing to the lack of accurate and tissue-specific biomarkers of liver fibrogenesis (Friedman and Pinzani 2022). Novel quantitative and non-invasive biomarker of HSC activation would provide an important tool for rapid assessment of therapeutic intervention, by allowing repeated assessment of disease activity in liver.

Positron Emission Tomography (PET) is a clinical imaging technique that uses positron emitting radionuclides incorporated into ligand for targeted detection of e.g. receptors or enzymes in living tissue. PET has excellent sensitivity and can detect attomolar concentration of receptors although spatial resolution is limited to millimeter range. Several targets have been proposed as suitable markers of activated HSCs to target by PET, including the platelet derived growth factor beta (PDGFRβ) (Friedman and Pinzani 2022; Wegrzyniak et al. 2021). The crucial requirements for PET technology in assessing liver fibrogenesis are as follows; the target must be specific for activated HSCs; the targeting ligand must have high affinity and selectivity for its target and the ligand must allow fast radiolabeling while maintaining binding capacity and stability in vivo. If all these requirements are fulfilled, the PET signal arising from the liver after injection of the radiopharmaceutical should be proportional to the amount of target on activated HSCs, and thus strongly associated to the activity of fibrotic disease (Supplementary Fig. 1A).

The Affibody molecule ATH001 (also known as Z_09591_) was developed for optimal affinity and selectivity towards PDGFRβ (Lindborg et al. 2011). Affibody molecules are small proteins of approximately 7 kDa size, which have many properties that make them ideal as PET tracer, including suitable size, fast clearance and targeting, high thermal stability, rapid extravasation into tissue enabling the use of short-lived radionuclides and potential for high affinity. Affibody molecule ATH001 in different formats, multimerizations and with different labels has recently been successfully validated for imaging of PDGFRβ in both tumors and liver (Wegrzyniak et al. 2023, 2024; Tolmachev et al. 2014; Strand et al. 2014; Li et al. 2024; Cai et al. 2023). Gallium-68 chelation using the established 2,2′,2″,2‴-(1,4,7,10-Tetraazacyclododecane-1,4,7,10-tetrayl) tetraacetic acid (DOTA) chelator is a common technique for PET labeling of peptides in the clinical setting. Gallium-68 labeling of DOTA conjugation of monomeric ATH001 Affibody molecule ([^68^Ga]Ga-DOTA-Cys-ATH001) was deemed the most straightforward and suitable format for clinical development (Supplementary Fig. 1B).

Here, we first use single cell sequencing to demonstrate that PDGFRβ is exclusively expressed on activated HSCs in both mice and humans, validating it as an appropriate target for PET imaging of fibrogenesis. Then, we have performed a detailed pharmacological characterization of [^68^Ga]Ga-DOTA-Cys-ATH001 as the last steps before clinical translation and human PET studies.

## Material and methods

### Pdgfrβ expression in liver cells in a mouse model of MASH

The single cell transcriptome of hepatic cells from a mouse disease model of liver fibrosis (Fibrosis and Tumor (FAT) MASH) and healthy control mice was investigated by single nucleus ribonucleic acid sequencing (snRNAseq). The dataset and the methodology were presented in detail previously (Wang et al. 2023; Yashaswini et al. 2024). Briefly, the FAT-MASH model is induced by a high fat, cholesterol and sugar diet in combination with weekly low-dose intraperitoneal administrations of carbon tetrachloride (CCl_4_) (Supplementary Fig. 2A) (Yashaswini et al. 2024; Tsuchida et al. 2018), while the healthy control mice were fed normal chow.

Here, the snRNAseq expression data on *Pdgfrbβ* from the aforementioned dataset (24-week FAT-MASH liver) is presented in detail, in addition to the endogenous ligands of PDGFRβ as well as other suggested imaging biomarker targets for detection of activated HSCs: integrins α_v_β_3_ and α_v_β_6_ (Wegrzyniak et al. 2021).

### PDGFRβ expression in liver cells in human individuals with MASH and healthy controls

A snRNAseq dataset on human liver biopsies from individuals with MASH and healthy individuals was reported in detail previously (Wang et al. 2023; Yashaswini et al. 2024). Here, the single nucleus expression data on *PDGFRβ* in the human liver tissue is presented, as well as the additional markers listed above.

### DOTA-Cys-ATH001 interferes with PDGF-bb activation of PDGFRβ

#### Cell culturing

Human foreskin primary fibroblasts AG1523 (Coriell Cell Repositories, USA) and human hepatic stellate cell lines LX-2 (SCC064, Sigma) (Xu et al. 2005) were cultured in at 37 °C in 5% CO_2_ humidified atmosphere in Dulbecco’s Modified Eagle’s medium (DMEM) (Sigma-Aldrich) supplemented with 10% or 2% bovine fetal serum (FBS) (Biowest) correspondingly. Cells were starved in DMEM, supplemented with 0.1% FBS for 5 h or overnight. Cell monolayers were stimulated with 5 ng/ml for 15 min for activation assay and with 20 ng/ml PDGF-BB (Chiron Corp.) for internalization assay.

#### Reagents and antibodies

Primary antibodies for immunoprecipitation of PDGFRβ (ctβ) were homemade, raised against a GST fusion of the C-terminal part of PDGFRβ (Karlsson et al. 2006) and the PDGFRβ antibody for immunoblotting were from R&D (AF385). The antibody against PDGFRα were from R&D (AF307-NA) and transferrin receptor (ab84036) from Abcam. Secondary antibodies for immunoblotting were HRP-conjugated anti-goat IgG (ThermoScientific) and mouse anti-rabbit IgG (Jackson ImmunoResearch).

#### Immunoprecipitation and immunoblotting

Cell monolayers were starved and stimulated with PDGF-BB at 50% confluency, washed twice in ice-cold phosphate-buffered saline (PBS) and lysed in RIPA buffer (0.5% deoxycholate, 0.1% SDS, 1% Triton X-100, 10% glycerol, 20 mM Tris, pH 7.4, 150 mM NaCl), supplemented with Halt protease and phosphatase inhibitors cocktail (ThermoScientific). Cell lysates were centrifuged at 13,000 rpm for 15 min at 4 °C, divided into two fractions and incubated with PDGFRβ or PDGFRα primary antibody, respectively, overnight at 4 °C. This followed by incubation with Rec-protein G-sepharose 4B conjugate (ThermoScientific), three washes in lysis buffer and elution of immunoprecipitated proteins. Samples were then subjected to SDS–polyacrylamide gel electrophoresis (SDS-PAGE) and electro-transferred to PVDF membranes (Immobilon). The membranes were blocked in 5% bovine serum albumin (BSA) in PBS, 0.05% Tween-20 and incubated at 4 °C overnight with primary antibodies, followed by three washes in PBS, 0.05% Tween-20 and incubation with HRP-conjugated secondary antibodies for 1 h at room temperature, followed by washing three more times. The proteins were visualized with the enhanced chemiluminescence (ECL) detection system on a charge-coupled device (CCD) camera (BioRad) and quantified using Bio-Rad ImageLab 6.0.1 software.

#### Cell surface internalization assay

Cell monolayers were starved overnight and stimulated with 20 ng/ml PDGF-BB at the indicated periods of time, following incubation with 0.2 mg/ml Sulfo-NHS-SS-Biotin (Pierce) in PBS for 1 h at 4 °C to label cell surface proteins. The excess of biotin was neutralized with 50 mM Tris (pH 8.0), then cells were washed in ice-cold PBS and lysed in RIPA buffer. Lysates were incubated with high-capacity streptavidin agarose resin (ThermoScientific) for 1 h at 4 °C, washed three times, eluted and resolved by SDS-PAGE, followed by immunoblotting as described above.

### In vitro autoradiography binding studies

Frozen biopsies of human liver (n = 10) with different degree of pathology-confirmed fibrosis were obtained from the Uppsala Biobank (#827). The Swedish Ethical Review Authority (2019-02790) approved the use of biobanked human biopsies for autoradiographic binding studies.

Sectioned U87 cell pellets were added to the assay as positive, PDGFRβ expressing controls. Frozen tissues from previous used preclinical models of liver fibrosis were also evaluated for binding of [^68^Ga]Ga-DOTA-Cys-ATH001: liver sections with different degrees of active fibrosis were evaluated, either healthy liver, liver from mice treated by CCl_4_ for 6 weeks, or liver from mice following two weeks regeneration following stopping CCl_4_ treatment. Frozen sections from two other preclinical models were also assayed: lung and control tissues from rats with bleomycin induced pulmonary fibrosis, as well as tumor and control tissues from a syngeneic murine MC38 colon carcinoma tumor model.

Briefly, frozen tissue sections (20 µm thickness, on Superfrost + objects glasses) were thawed and equilibrated in incubation buffer (PBS + 1% BSA) for 10 min at RT with or without addition of 1.2 µM unlabeled DOTA-Cys-ATH001. Then, [^68^Ga]Ga-DOTA-Cys-ATH001 was added corresponding to a molar concentration of 5 nM, followed by incubation at RT for 60 min. Next, the sections were washed two times in PBS + 1%BSA for 1 min, one time in PBS and quickly dipped in MilliQ water. The sections were dried at 37 °C for 10 min and then exposed against a phosphorimager plate overnight. A reference was added (10 µl of incubation buffer) to enable quantification. The plate was digitalized using a Typhoon phosphorimager scanner (GE Healthcare). The sections themselves, or sequential sister sections were stained for collagen (Sirius Red, SIR) or for PDGFRβ as described below. The autoradiography images were quantified in ImageJ (NIH), to assess the total and non-specific binding as fmol/mm^3^ in each section. Similarly, the histology sections were analysed for e.g. percent positive area of SIR (in ImageJ) and correlated to the autoradiography binding data.

### Animal handling and housing

Mice were housed at five per cage and had access to food and water ad libitum*.* They were kept under a constant temperature of 22 °C and humidity (50%), in a 12 h light/12 h dark rhythm in individually ventilated cages. The pig was acquired from the breeder on the morning of the experiment. The Animal Ethics Committee of the Swedish Animal Welfare Agency approved all experimental protocols. The procedures were performed in agreement with the ARRIVE recommendations and institutional guidelines (“Uppsala University guidelines on animal experimentation”, UFV 2007/724).

### In vivo proof of principle of PDGFRβ detection demonstrated in a U87 xenograft mice model

BALB/C immunodeficient mice (nu/nu, female, 19–20 g, n = 10) were induced with hind leg xenografts by subcutaneous implantation of U87 cells (2 million cells/mouse). After 4–6 weeks, mice were examined by [^68^Ga]Ga-DOTA-Cys-ATH001, in order to evaluate the in vivo targeting of a PDGFRβ positive lesion. The mice used for ex vivo organ distribution (n = 8) were injected intravenously with a target dose of 1 MBq [^68^Ga]Ga-DOTA-Cys-ATH001 (corresponding to approximately 0.3 µg peptide mass, based on the results of the dose finding study). Half of the group of mice (n = 4) were pre-injected with 1 mg/kg Cys-ATH001 to pre-block the available PDGFRβ. The mice were euthanized 60 min after injection, and organs (blood, lungs, liver, spleen, kidneys, tumor, muscle, bone and gastrointestinal (GI) tract) excised, weighed and measured for radioactivity in a gamma counter. Uptake was expressed as %ID/g.

The remaining two mice were injected a target dose of 10 MBq to allow PET scanning, of which one received a pre-injection of 1 mg/kg Cys-ATH001. The mice were anaesthetized by sevoflurane and examined by PET/MRI 45 min after injection, with a 30 min static scan. For all mice, the U87 tumor, spleen and muscle were fixed in formalin and embedded in paraffin for histological analysis (e.g. staining for SIR and MTC, immunostaining for PDGFRβ, see below).

### Biodistribution and in vivo stability in rat assessed by PET/MRI

The biodistribution (n = 3) and in vivo stability (n = 4) of [^68^Ga]Ga-DOTA-Cys-ATH001 was evaluated in healthy Sprague Dawley rats.

For the PET biodistribution study, rats (male, n = 3, 352–375 g) were anaesthetized by sevoflurane and placed in the gantry of a preclinical PET/ MRI scanner (nanoPET/MRI, 3T, Mediso, Hungary). A catheter was placed in the lateral tail vein, and administered a target dose of 20 MBq [^68^Ga]Ga-DOTA-Cys-ATH001. The PET examination was started at the same time as the injection. The rat was examined with a multi-pass sequence, in order to capture the dynamic biodistribution in the entire body despite the axial field of view being only 10 cm. Briefly, the sequence was designed to perform passes of three bed positions with increasing duration per bed position as time passed from injection (Pass 1 and 2: 100 s per bed position, 5 min in total. Pass 3–4: 200 s per bed position, 10 min in total. Pass 5–7: 600 s per bed position, 30 min in total). The total time for the sequence was 120 min and the animal was the euthanized. Antomical axial MRI scans over the entire body was acquired postmortem.

Co-registered PET/MRI images were segmented for important tissues (brain, large intestines, small intestines, stomach contents, myocardium, heart content (left ventricle), kidneys, liver, lungs, muscle, red marrow (bone marrow), bone tissue, spleen, urinary bladder content) using PMOD 4.0 (PMOD Technologies). Diffuse organs such as pancreas were not segmented but were not expected to have high uptake anyway based on visual inspection. Uptake in individual tissues over time was expressed as Standard Uptake Values (SUV).

A further n = 4 rats were administered 30–40 MBq of [^68^Ga]Ga-DOTA-Cys-ATH001 to analyse in vivo stability of the tracer at different time points after injection: 60 min (n = 2) or 120 min (n = 2). The animals were euthanized under anesthesia, blood taken by heart puncture, which was then centrifuged (3000×*g* 2 min, 4 °C) to separate the plasma. Plasma proteins were precipitated by adding acetonitrile and the percentage of radioactivity associated to intact DOTA-Cys-ATH001 was analyzed for each sample by HPLC, using the same processing and methods as for in vitro plasma stability analysis described in the Supplementary Methods.

### Biodistribution in pig assessed by PET/CT

To verify the biodistribution observed in mice and rats in a larger animal model with higher fidelity to human physiology, a PET biodistribution study was also performed in a pig.

Briefly, a Swedish landrace pig (male, 2 months old and weighing 33 kg) was sedated by an intramuscular injection of tiletamine-zolazepam (Boehringer Ingelheim). Continuous anesthesia was maintained by intravenous infusion of ketamine 20 mg/kg/h (Ketaminol), fentanyl 5 mg/kg/h (Pharmalink) and pancuronium 0.24 mg/kg/h (Pavulon, Organon Teknika), after intubation and ventilation. Standard monitoring was performed as previously described (Wegrzyniak et al. 2023). The pig was placed on its back in the gantry of a PET/CT scanner (Discovery MI, 25 cm Field of View, GE Healthcare). First, high dose CT scans were acquired for attenuation correction and anatomical co-registration of PET images. [^68^Ga]Ga-DOTA-Cys-ATH001 (29.6 MBq, corresponding to approximately 17 µg peptide mass, ≈0.5 µg/kg) was injected intravenously as a bolus. A dynamic multibed PET protocol using repeated whole-body passes was started at the time of injection, designed to capture the biodistribution in the entire body over time by overcoming the limited FoV of the scanner, similarly as for the rat study above. Firstly, a dynamic 15-min scan was acquired over the level of the heart to capture the blood kinetics. Secondly, whole body passes were performed as follows: 5 bed positions in total, first 5 passes using a duration of 120 s/bed, then 4 passes with 300 s/ bed – in total 150 min. The actual duration of the entire scan was 166 min, due to time losses when switching from the initial heart scan to the whole-body sequence, and when manually restarting the whole-body pass protocol after each pass.

After the scanning, the pig was euthanized under deep anesthesia by intravenous KCl.

PET/CT images were analyzed using PMOD 4.0 (PMOD Technologies), segmenting the same organs (but adding e.g. gallbladder) as for the rat PET/MRI scans described above, and expressed as Standard Uptake Values (SUV). 3D PET images were acquired by the Carimas software (Turku PET Center).

### Statistics

Data was visualized and analysed using GraphPad Prism 10. Differences between groups were performed using Students T-test or, for multigroup analysis, ANOVA.

## Results

### Pdgfrβ is selectively expressed by activated HSCs in mouse and human liver

Bulk RNA sequencing demonstrated ≈tenfold increased expression of *Pdgfrβ* mRNAfrom liver of FAT-MASH compared to control mice (Supplementary Fig. 2B).

**Fig. 1:**
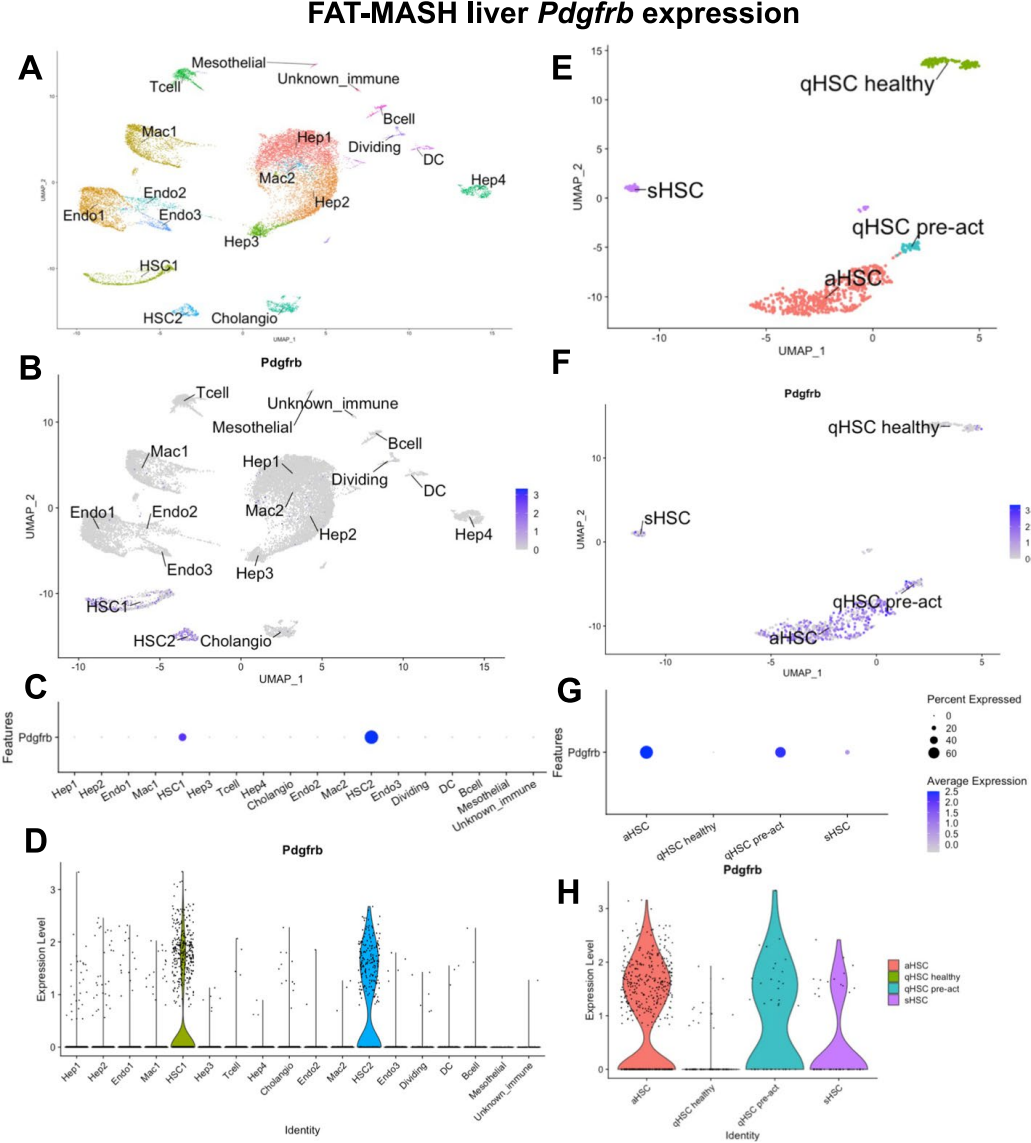
snRNAseq analysis of *Pdgfrb* transcription in liver of FAT-MASH mice. Clustering of snRAseq data (**A**). *Pdgfrb* expression was only seen in clusters identified as HSCs (HSC1 and HSC2) (**B**). Percent of cells in each cluster that expressed *Pdgfrb* (**C**), and expression level in individual cells in each cluster (**D**). Re-clustering of the HSC1 and HSC2 clusters demonstrated sub-populations of HSCs: quiescent HSCs (qHSC healthy), quiescent precursors of activated HSCs (qHSC pre-act), activated HSCs (aHSCs) and senescent HSCs (sHSCs) (**E**). PDGFRβ expression was mainly found in aHSCs and qHSC pre-act, with lesser or negligible expression in sHSCs and qHSC healthy, respectively (**F**–**H**)

snRNAseq of FAT-MASH mouse liver further demonstrated that *Pdgfrβ* was almost exclusively expressed on cells assigned to clusters corresponding to HSCs (Fig. 1A-D). The expression on other cell types e.g. hepatocytes, endothelial cells, and immune cells (including macrophages) was negligible.

Four distinct cell populations were identified when re-clustering the HSCs: quiescent HSCs (qHSC), quiescent precursors of activated HSCs (qHSC pre-act), activated HSCs (aHSCs) and senescent HSCs (sHSCs) (Fig. 1E). *Pdgfrb* was mainly seen in aHSCs where more than 60% of the cells demonstrated high expression (Fig. 1F-H). Approximately 40% of the qHSC pre-act cells also expressed *Pdgfrβ*. Expression of *Pdgfrβ* in sHSCs was minor (< 10%) and negligible in qHSC.

Futhermore, snRNAseq identified primarily hepatic macrophages (Kupffer cells), and to a lesser extent endothelial cells and cholangiocytes, as the main sources of the endogenous PDGFRβ ligand PDGF-B (Supplementary Fig. 4A, B). On the other hand, cholangiocytes and to a lesser extent endothelial cells were the main sources of the second important PDGFRβ ligand PDGF-D (Supplementary Fig. 4C, D).

A similar result was obtained from human liver samples from individuals with MASH and healthy individuals, where *PDGFRβ* expression was specific for the HSC cluster (Supplementary Fig. 5A–C). Re-clustering of HSCs further showed that high *PDGFRβ* expression was specific for aHCSs and deactivated HSCs (dHSCs), while essentially absent from qHSCs (Supplementary Fig. 5D–F). The whole liver clusters and the HSCs sub-clustering could be further divided into cells from different disease states, where *PDGFRβ* expression was primarily found in HCSs in individuals with MASH (Supplementary Fig. 5G), and in these individuals more specifically aHSCs and sHSCs (Supplementary Fig. 5H).

The sources of mRNA encoding endogenous PDGFRβ ligands *PDGFB* (Supplementary Fig. 6A, B) and *PDGFD* (Supplementary Fig. 6C, D) were less cell population-specific in humans than in mouse, but endothelial cells and cholangiocytes, respectively, were identified as important contributors.

The transcripts for the integrin subunits α_v_ (*ITGAV*) or β_6_ (*ITGB6*) were not selectively expressed in HSCs and were instead found on many other liver cell populations in both mouse FAT-MASH (Supplementary Fig. 7A–C) or human MASH (Supplementary Fig. 7D–F). The integrin subunit β_3_ (*ITGB3*) was more selective for HSCs in mouse but not human liver (Supplementary Fig. 7D–F). Additionally, *ITGB3* expression was primarily assigned to quiescent precursors of activated HSCs and senescent HSCs in mouse liver (Supplementary Fig. 7B).

### Affinity and selectivity of DOTA-Cys-ATH001 towards PDGFRβ by SPR

The resulting interaction kinetics represent three separate experiments with new immobilizations and new sample dilutions. The resulting kinetics was very reproducible. For DOTA-Cys-ATH001 the K_D_ was 282 ± 28 pM (n = 3) for rhPDGFRβ and 2.25 ± 0.26 nM (n = 3) for rmPDGFRβ (Supplementary Fig. 8A, B and Table 1). For rhPDGFRα no interaction between the receptor and DOTA-Cys-ATH001 could be detected at concentration up to 3000 nM (Supplementary Fig. 8C).

**Table 1:** Results of the kinetic analysis of ATH001 Affibody molecules interacting with human or mouse recombinant PDGFRß

Interaction towards PDGFRß		K_D_ (nM)	*k*_*a*_ (1/Ms)	*k*_*d*_ (1/s)
Human PDGFRβ	Average	0.28	4,660,000	0.0013
	SD	± 0.03	± 636,000	± 4.99E-05
	n	6	6	6
Mouse PDGFRβ	Average	2.3	4,360,000	0.0097
	SD	± 0.3	± 928,000	± 0.0016
	n	4	4	4

rhPDGFRβ at 100 nM interacted with rhPDGF-BB immobilized at the surface (Supplementary Fig. 8D). Co-injection with increasing amounts of DOTA-Cys-ATH001 competed dose-dependently for binding to rhPDGF-BB, seen as a progressive decrease in response, as rhPDGFRβ becomes unavailable for binding with increasing amounts of DOTA-Cys-ATH001. The IC_50_-values for competition with rhPDGF-BB for binding to rhPDGFRβ was in the range of 45–60 nM (n = 2) (Supplementary Fig. 8E).

### DOTA-Cys-ATH001 interferes with PDGF-BB activation of PDGFRβ

**Fig. 2:**
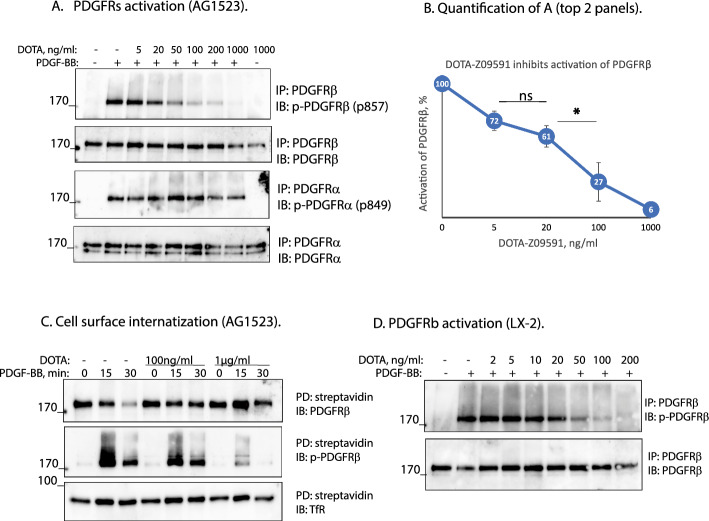
In vitro PDGFRβ activation. **A** AG1523 fibroblasts were starved for 5 h and stimulated with 5 ng/ml PDGF-BB, premixed in the media with increasing concentrations of DOTA-Cys-ATH001 (labeled “DOTA” in the figure). Total lysates were immunoprecipitated independently for PDGFRβ or PDGFRa with receptor-specific antibodies. Eluates were immunoblotted for phosphorylated PDGFRβ (top panel) or PDGFRa (third panel) and reblotted for total amounts of PDGFRβ (second panel) or PDGFRa (bottom panel). **B** Quantification of activation of PDGFRβ as presented in (**A**) based on 3 independent repeats. The amount of activated receptor was adjusted to the amount of total receptor, thereafter activation at stimulation with PDGG-BB but without addition of DOTA-Cys-ATH001 (second line in panel (**A**)) was set as 100% of activation of the receptor and decrease of activation with the addition of DOTA-Cys-ATH001 was calculated relevant to that. * = *p* < 0.05. **C.** Internalization of PDGFRβ from the cell surface. AG1523 fibroblasts were starved overnight and stimulated with 20 ng/ml of PDGF-BB for 15 or 30 min to promote internalization of PDGFRβ from the cell surface; 0 min represents unstimulated cells. DOTA-Cys-ATH001 was added simultaneously with PDGF-BB at indicated concentrations; the corresponding unstimulated cells were untreated or treated with DOTA-Cys-ATH001 alone for 30 min. Cells were then incubated with sulfo-NHS-SS-biotin in order to label PDGFRβ that remained on the cell surface after stimulation. Biotinylated PDGFRβ was precipitated with streptavidin agarose from the total cell lysates and immunoblotted for total amount of PDGFRβ (top panel), phosphorylated PDGFRβ (middle panel) and transferrin receptor (TfR) (bottom panel) as a control. **D.** The activation of PDGFRβ as presented in (A) was repeated on immortalized human liver hepatic stellate cells (LX-2). Phosphorylation of PDGFRβ (top panel) in response to stimulation with PDGF-BB without or with addition of DOTA-Cys-ATH001 and the total amount of immunoprecipitated PDGFRβ (bottom panel) are presented. IP: immunoprecipitation; IB: immunoblotting; PD: pull-down

DOTA-Cys-ATH001 alone does not activate PDGFRβ at any of the concentrations tested. On the contrary, it is able to inhibit activation of PDGFRβ at doses above 20 ng/ml, as detected by immunoblotting for phosphorylation of Tyr857, which is positioned in the activation loop of the kinase domain of PDGFRβ and represents a reliable readout of PDGFRβ activation (Tyr849 for PDGFRα). We observed that increasing concentrations of DOTA-Cys-ATH001 that was added to the cells together with PDGF-BB led to a dose-dependent decrease of PDGFRβ activation by PDGF-BB (Fig. 2A-B). This suggests that DOTA-Cys-ATH001 may interfere with PDGF-BB-induced activation of PDGFRβ, while binding of DOTA-Cys-ATH001 alone to the receptor does not have any effect. Consistent with this, we observed that simulation of AG1523 fibroblasts with PDGF-BB and increasing concentrations of DOTA-Cys-ATH001 delayed internalization of PDGFRβ from the cell surface (Fig. 2C) that normally follows the activation of the receptor. The effect of DOTA-Cys-ATH001 on the activation of PDGFRβ was reproduced in immortalized human hepatic stellate cells LX-2 (Fig. 2D). Interestingly, we observed that DOTA-Z09591 binds specifically to PDGFRβ while being unable to affect activation of PDGFRα with PDGF-BB in the same cells (Fig. 2A).

### Radiolabeling optimization and in vitro and plasma stability of [^68^Ga]Ga-DOTA-Cys-ATH001

[^68^Ga]Ga-DOTA-Cys-ATH001 demonstrated optimized radiolabeling yield and purity at 15 min incubation at 70 °C, and increasing temperature further did not improve yield or purity (Supplementary Fig. 9A, B). Purity was consistently > 95% after purification, and molar activity was reproducibly in the range of 10–20 MBq/nmol, sufficient for in vitro and in vivo preclinical evaluation. [^68^Ga]Ga-DOTA-Cys-ATH001 demonstrated suitable stability and shelf-life for at least 1 h (Supplementary Fig. 10A, B). Challenge with EDTA did not impair purity, indicating stable chelation of Gallium-68 in DOTA (Supplementary Fig. 10A, B).

[^68^Ga]Ga-DOTA-Cys-ATH001 was stable in both human and rat plasma at 37 °C in vitro, with more than 90% and 85% intact after 60 and 120 min, respectively (Supplementary Fig. 11A, B).

### In vitro binding of [^68^Ga]Ga-DOTA-Cys-ATH001 to U87 cells

[^68^Ga]Ga-DOTA-Cys-ATH001 bound cultured viable U87 cells in a dose-dependent manner and could be completely inhibited by addition of 16 µM unlabeled DOTA-Cys-ATH001, demonstrating low non-specific binding (Supplementary Fig. 12). The binding was saturable with increasing concentration of [^68^Ga]Ga-DOTA-Cys-ATH001, and the dissociation constant was estimated to be in the low nanomolar range (K_d_ = 3.7 nM) (Supplementary Fig. 12).

### Autoradiography assay using human and murine liver sections with different stages of liver fibrosis

**Fig. 3:**
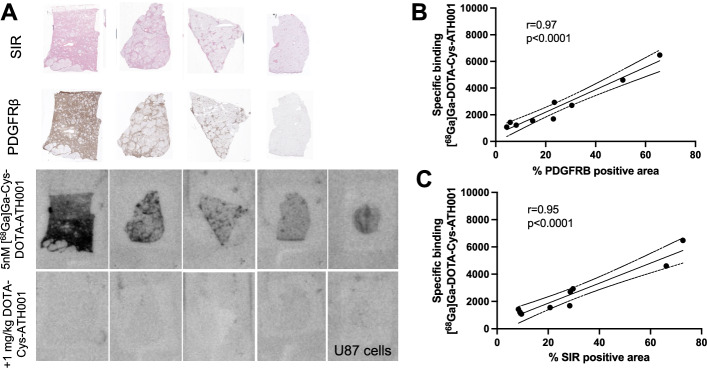
Representative autoradiograms showing binding of [^68^Ga]Ga-DOTA-Cys-ATH001 to human liver sections (from a total of n = 9 donors) with variable degree of fibrosis (as assessed by SIR staining) and PDGFRβ immunopositivity, either tracer alone or co-incubation with Cys-ATH001 in excess (**A**). Correlation between specific binding in human fibrotic liver sections, and PDGFRβ positive area (**B**). Correlation between specific binding in human fibrotic liver sections, and SIR positive area (**C**)

[^68^Ga]Ga-DOTA-Cys-ATH001 was evaluated for binding sections of human frozen liver biopsies from individuals with variable amount of hepatic fibrosis, as well as PDGFRβ positive sectioned U87 cells as positive controls. [^68^Ga]Ga-DOTA-Cys-ATH001 binding intensity was high in human liver tissues with high fibrosis score and conversely low in tissues with low fibrosis score (Fig. 3A). The binding pattern in each biopsy demonstrated overlap with areas positive for PDGFRß immunostaining and collagen fibers. Importantly, the binding was abolished by co-incubation with excess (1 µM) unlabeled Cys-ATH001, further indicating that the binding was saturable and receptor-specific (Fig. 3A). The specific binding of [^68^Ga]Ga-DOTA-Cys-ATH001 correlated strongly with both the percentage of PDGFRβ immunopositive tissue in each tissue (Fig. 3B) as well as the fibrosis degree as assessed by collagen (SIR) positive area (Fig. 3C).

In vitro autoradiography of sections of mouse liver demonstrated higher binding of [^68^Ga]Ga-DOTA-Cys-ATH001 to livers with CCl_4_-induced fibrogenesis (Supplementary Fig. 13) than in healthy controls (Supplementary Fig. 13A). The increased tracer binding was localized to the hepatic sinusoids, which were recognized by immunostaining for PDGFRβ and SIR in animals with induced liver fibrosis (Supplementary Fig. 13B). Binding of [^68^Ga]Ga-DOTA-Cys-ATH001 was normalized in liver sections after two weeks regression of fibrosis, consistent with the decreased PDGFRβ immunopositivity (Supplementary Fig. 13C). [^68^Ga]Ga-DOTA-Cys-ATH001 binding thus displayed sensitivity to rapid changes in PDGFRβ expression and active fibrosis in tissue.

### Biodistribution and detection of PDGFRβ positive lesions by [^68^Ga]Ga-DOTA-Cys-ATH001 PET in mice

First, the optimal in vivo dosing of [^68^Ga]Ga-DOTA-Cys-ATH001 was evaluated in healthy mice using PDGFRβ positive spleen as a positive control tissue. Given the picomolar affinity of DOTA-Cys-ATH001 to PDGFRβ, there is a risk for mass effect and self-blocking at higher doses of injected peptide. [^68^Ga]Ga-DOTA-Cys-ATH001 demonstrated low blood signal (as %ID/g) and mainly renal clearance (Supplementary Fig. 14). Splenic binding was high at low tracer mass doses corresponding to up to 0.3 µg (or 12 µg/kg). Mass effect, or self-blocking, was seen already at peptide doses of 1.4 µg (56 µg/kg), consistent with the high affinity DOTA-Cys-ATH001 (Supplementary Fig. 14). Complete blocking of the splenic binding was achieved at 8 mg/kg.

Next, the performance of [^68^Ga]Ga-DOTA-Cys-ATH001 in an in vivo U87 xenograft model was evaluated, to provide proof of concept that the tracer can detect PDGFRβ immunopositive lesions. [^68^Ga]Ga-DOTA-Cys-ATH001 binding in the tumor was clearly visible (7.9 ± 0.8%ID/g) (Supplementary Fig. 15A, B), and the signal could be almost completely abolished by pre-treatment with Cys-ATH001 in excess (2.6 ± 0.5%ID/g). Similar blockable binding was also seen in spleen, which was confirmed to be immunopositive for PDGFRβ, as was the explanted tumor (Supplementary Fig. 15C).

### Biodistribution, plasma concentration, in vivo stability and dosimetry of [^68^Ga]Ga-DOTA-Cys-ATH001 in rat and pig

The biodistribution of [^68^Ga]Ga-DOTA-Cys-ATH001 was evaluated in healthy rats and pig by PET. Furthermore, the in vivo stability of [^68^Ga]Ga-DOTA-Cys-ATH001 was evaluated in blood samples from rats injected with the tracer.

**Fig. 4:**
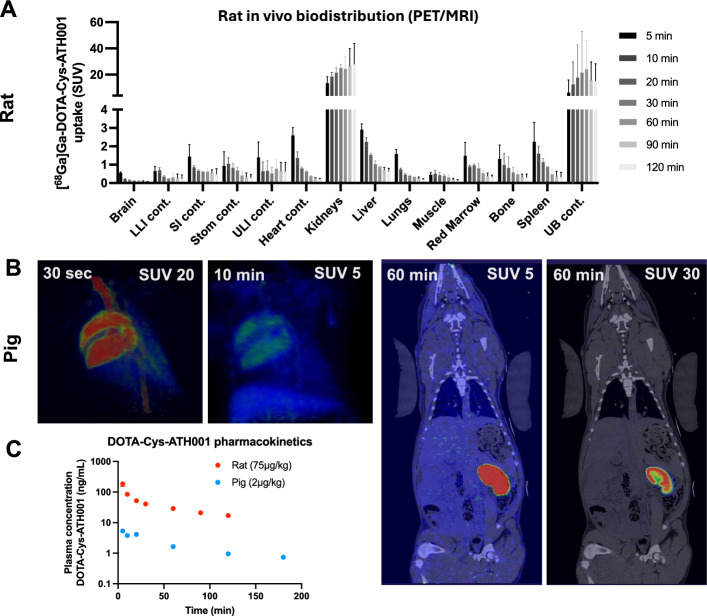
Biodistribution of [^68^Ga]Ga-DOTA-Cys-ATH001. Tissue uptake of [^68^Ga]Ga-DOTA-Cys-ATH001 over time in rats (n = 3), quantified from PET/MRI images Representative PET/MRI images of tissue uptake in rats (**A**). Representative PET/MRI images of tissue uptake in pig (n = 1), at early time points over heart (left panels) and after 1 h (right panels, whole body scan, coronal projections) (**B**). Pharmacokinetics of in blood at different doses in different species (**C**)

In rat [^68^Ga]Ga-DOTA-Cys-ATH001 was excreted almost exclusively by the kidney, demonstrating retention in the renal cortex in combination with accumulation in the urine (Fig. 4A). [^68^Ga]Ga-DOTA-Cys-ATH001 was rapidly cleared from most other heathy tissues, demonstrating low background. [^68^Ga]Ga-DOTA-Cys-ATH001 was stable in rat plasma in vivo with around 70% of radioactivity associated to intact tracer after 60 min, and 65% after 120 min (Supplementary Fig. 16A, B).

Biodistribution in pig was similar to that in rat, demonstrating rapid biodistribution after intravenous injection (Fig. 4B), followed by rapid clearance and washout from most tissues including liver and the blood pool. Excretion was almost completely renal, as seen by trapping in the renal cortex at delayed imaging timepoints (Fig. 4B).

The plasma concentrations of DOTA-Cys-ATH001 in blood was estimated from the radioactive signal and the molar activity to convert MBq to nanograms per milliliter. DOTA-Cys-ATH001demonstrated rapid washout during the first three hours after injection (Fig. 4E). In pig, where a total dose of approximately 60 µg (2 µg/kg) was injected, the peak concentration was less than 10 ng/mL. After two hours, the plasma concentration of DOTA-Cys-ATH001 had fallen to below 1 ng/mL.

The predicted human dosimetry profile was extrapolated from both in vivo rat and pig data.

The human predicted dosimetry extrapolated from rat biodistribution indicated an effective clinical dose of [^68^Ga]Ga-DOTA-Cys-ATH001 of 15.9 µSv/MBq, while extrapolation from pig data indicated an effective dose of 12.9 µSv/MBq. In order to limit the exposure to 10 mSv, the amount of administered radioactivity should thus be limited to 628 MBq. These dosimetry values are in range with those expected for Gallium-68 radiolabeled peptides of similar size. The intended clinical radioactive dose is in the range of 200–300 MBq, in order to obtain PET images with acceptable quality for detection of uptake in tissues. At a dose of 300 MBq [^68^Ga]Ga-DOTA-Cys-ATH001, the total effective dose would be 4.77 mSv for a PET scan (based on 15.9 µSv/MBq for rats).

### Microdosing acute toxicology study to determine NOAEL

Intravenous bolus administration of a single dose of DOTA-Cys-ATH001 at 10, 100 and 1000 µg/kg in rats had little effect on body weight, clinical health status and food consumption. No marked changes in haematology or coagulation parameters were noted. A minimal, but significant increase in serum albumin, amylase and total protein was evident in high dose animals 24 h after administration. In addition, there were decreases in thymus, liver, kidney, lung and spleen weight. Importantly, there were no changes in clinical chemistry parameters or organ weights in recovery animals, indicating that the effects seen at 24 h were transient. A transient increase of minimal to mild perivascular infiltration of mixed inflammatory cells were observed in the lungs of high dose animals. Overall, the documented rapid and complete reversibility of the described clinical chemistry, organ weight and lung changes observed in high dose animals (1000 µg/kg) suggest that this dose is the NOAEL.

### NOAEL and Pharmacokinetics of DOTA-Cys-ATH001

DOTA-Cys-ATH001 demonstrated an excellent safety profile at all doses tested in rat and NOAEL was thus established to be at least 1000 µg/kg. Quantifiable plasma concentrations of DOTA-Cys-ATH001 were measurable after 5 min of treatment. In the group administered 100 µg/kg DOTA-Cys-ATH001, plasma concentration was below the detection limit after 15 min. The Cmax for this dose was on average 236.98 ng/mL. In the group administered 1000 µg/kg DOTA-Cys-ATH001, plasma concentration declined rapidly and was below the detection limit after 1 h. The Cmax for 1000 µg/kg DOTA-Cys-ATH001 administrated intravenously was on average 2874.61 ng/mL, the clearance was 0.75 L/h/kg and the Volume of distribution 0.36 L/kg.

## Discussion

Non-invasive technologies to quantify liver fibrosis are urgently needed to track the response to therapy as well as to identify patients with increasing fibrosis over time. MASH (formerly ‘NASH’) has traditionally required liver biopsy for definite diagnosis and fibrosis staging, but tissue biopsy can be associated with complications and sampling variability (Friedman and Pinzani 2022; Wegrzyniak et al. 2021). On the other hand, most current non-invasive clinical diagnostic tools detect the existing fibrotic scarring (e.g. Transient or Magnetic Resonance Elastography) but reveal limited information about activity or rate of ECM production and deposition. Blood markers of fibrogenesis are in development but are not tissue-specific and often suffer from lack of sensitivity (Sanyal et al. 2023).

Accordingly, we propose to use molecular imaging by PET technology of activated HSCs for non-invasive, tissue-specific detection of liver fibrogenesis.

The critically important role of PDGFRß in HSC biology has been known for decades (Tsuchida and Friedman 2017; Wong et al. 1994; Pinzani et al. 1996), but its quantitative expression pattern across subpopulations of HSCs has been poorly understood. Here, we confirm by single nucleus RNA sequencing that *Pdgfrß* mRNA is specifically expressed by HSCs in the context of liver fibrosis, with minimal expression on other cell populations, including hepatocytes and resident immune cells. Importantly, *Pdgfrß* expression is virtually absent on quiescent HSCs, but is upregulated specifically in activated HSCs as well as a smaller subpopulation defined as a quiescent precursor of activated HSCs. These novel findings support the rationale of measuring PDGFRß content in the liver, as a surrogate marker of HSC activity and subsequently collagen deposition. By contrast, other proposed targets for detection of HSCs (integrins α_v_β_3_ and α_v_β_6_) were much less selectively expressed and were present also in other cell populations in the liver.

[^68^Ga]Ga-DOTA-Cys-ATH001 is an Affibody molecule that is being developed for molecular imaging of PDGFRß. ATH001 binds to the extracellular region of the PDGFRß monomer which is easily accessible to peptide and protein-based tracers. The efficacy of PDGFRß PET for detecting active liver fibrosis has recently been demonstrated preclinically using analogues of ATH001 labeled with various radionuclides including fluorine-18 (Wegrzyniak et al. 2023, 2024; Li et al. 2024), but neither of those constructs are ideal for clinical translation due to considerations on peptide precursor CMC, GMP radiochemistry or dosimetry. Gallium-68 and DOTA chelation radiochemistry, on the other hand, is widely available across most PET sites and there are decades of experience including safety data. A larger version of ATH001 has previously been labeled with Gallium-68 and evaluated for binding to PDGFRß-expressing xenografts by Strand et al. (Strand et al. 2014). That analogue comprised the ATH001 binding motif (58 amino acids), as well as restriction enzyme sites and a histidine-tag, bringing the total size to 78 amino acids. For clinical use, a minimized construct is preferred to enable rapid biodistribution and clearance, which is composed of solely the binding sequence ATH001 as well as a linker and a DOTA chelator. Thus, we selected [^68^Ga]Ga-DOTA-Cys-ATH001 as the most suitable analogue for future use in the clinical setting.

Here, we present a detailed pharmacological characterization of [^68^Ga]Ga-DOTA-Cys-ATH001 in preparation for clinical translation. We show that DOTA-Cys-ATH001 binds to human PDGFRß with picomolar affinity. Furthermore, DOTA-Cys-ATH001 acts as an antagonist and compete dose-dependently with endogenous PDGF-BB for binding to PDGFRß with an IC_50_ of approximately 45–60 nM (recombinant protein, SPR) or 30–40 ng/mL (cells, activation assay), but does not itself elicit phosphorylation at any dose.

[^68^Ga]Ga-DOTA-Cys-ATH001 demonstrated specific targeting of PDGFRß-positive fibrotic lesions in liver tissue sections from both human and mice. Importantly, the tracer binding to human liver biopsies correlated linearly with the amount of PDGFRß, which is required for in vivo receptor quantitation by PET. [^68^Ga]Ga-DOTA-Cys-ATH001 binding in vitro and in vivo was receptor specific, as it could be abolished by unlabeled ATH001 in excess. [^68^Ga]Ga-DOTA-Cys-ATH001 performed with a similar efficacy in detecting active fibrotic liver lesions as both [^18^F]AlF-RESCA-Z09591 (Wegrzyniak et al. 2024) or [^18^F]TZ-Z09591 (Wegrzyniak et al. 2023).

[^68^Ga]Ga-DOTA-Cys-ATH001 demonstrated excellent shelf-life and plasma stability both in vitro and in vivo for up to 2 h, in line with similar Gallium-68 labeled DOTA-conjugated Affibody molecules. Further in vivo evaluation in rats and pig showed fast biodistribution followed by rapid clearance, resulting in low tissue background uptake in liver and most other tissues after around 1 h. Thus, high contrast PET imaging of PDGFRß-positive lesions across the entire body should be possible given the excellent binding affinity and sufficient stability of [^68^Ga]Ga-DOTA-Cys-ATH001. In vivo detection of PDGFRβ activity by [^68^Ga]Ga-DOTA-Cys-ATH001 PET can thus have widespread utility for detection of key fibrogenic cell populations also in other indications, such as lung fibrosis, heart fibrosis and tumor stroma. Kidney was the sole route of excretion by design, to avoid any non-specific background uptake in the liver. However, this means that [^68^Ga]Ga-DOTA-Cys-ATH001 in its current iteration cannot be used to detect fibrogenesis in the kidney, given the very strong background.

The microdosing toxicology study determined that NOAEL for DOTA-Cys-ATH001 was at least 1 mg/kg, meaning that the intended clinical mass dose of around 1 µg/kg for future PET scans are safe for use, while conforming to microdosing regulatory guidelines. The rapid clearance and safety of DOTA-Cys-ATH001was further demonstrated by the pharmacokinetic assessments which showed that the plasma concentration was around 1 ng/mL an hour after dosing with 2 µg/kg [^68^Ga]Ga-DOTA-Cys-ATH001. Given that the IC_50_ was shown to be in excess of 30 ng/mL it is unlikely that a clinical PET dose would produce any sustained inhibition of PDGFRß.

Finally, the human predicted dosimetry of [^68^Ga]Ga-DOTA-Cys-ATH001 was in the expected range of similar Gallium-68 labeled peptides, enabling dosing of in excess of 600 MBq tracer without reaching common regulatory limits. Thus, based on preclinical dosimetry data [^68^Ga]Ga-DOTA-Cys-ATH001 could be dosed in the range of 200–300 MBq during repeated PET scans, enabling the possibility of monitoring anti-fibrotic interventions in the context of drug development or patient management. Dosing levels in this range is clearly sufficient for modern PET systems given their high sensitivity.

## Conclusion

Single nucleus RNA sequencing data demonstrated that *Pdgfrß* expression is restricted to activated HSCs in the fibrotic liver, and thus is a suitable imaging target for detection of active fibrogenesis.

Furthermore, the detailed pharmacological, pharmacokinetic and toxicological characterization of [^68^Ga]Ga-DOTA-Cys-ATH001 presented here indicates that it is a promising and safe PET imaging biomarker for detection of activated HSCs in the clinical setting. Future clinical studies will seek to link PDGFRß PET imaging to the stage of fibrosis and correlation with fibrosis content and disease progression.

## Supplementary Information

Additional file 1 (DOCX 6400 KB)

## Data Availability

The datasets used and/or analyzed during the current study are available from the corresponding author on reasonable request.

## References

[CR1] Cai H, Li Z, Shi Q, Yang H, Xiao L, Li M, et al. Preclinical evaluation of ^68^Ga-radiolabeled trimeric affibody for PDGFRβ-targeting PET imaging of hepatocellular carcinoma. Eur J Nucl Med Mol Imaging. 2023;50(10):2952–61. 10.1007/s00259-023-06260-x. 37256321 10.1007/s00259-023-06260-x PMC10382327

[CR2] Cogliati B, Yashaswini CN, Wang S, Sia D, Friedman SL. Friend or foe? The elusive role of hepatic stellate cells in liver cancer. Nat Rev Gastroenterol Hepatol. 2023;20(10):647–61. 10.1038/s41575-023-00821-z. 37550577 10.1038/s41575-023-00821-z PMC10671228

[CR3] Friedman SL, Pinzani M. Hepatic fibrosis 2022: unmet needs and a blueprint for the future. Hepatology. 2022;75(2):473–88. 10.1002/hep.32285. 34923653 10.1002/hep.32285 PMC12179971

[CR4] Higashi T, Friedman SL, Hoshida Y. Hepatic stellate cells as key target in liver fibrosis. Adv Drug Deliv Rev. 2017;121:27–42. 10.1016/j.addr.2017.05.007. 28506744 10.1016/j.addr.2017.05.007 PMC5682243

[CR5] Karlsson S, Kowanetz K, Sandin Å, Persson C, Östman A, Heldin C-H, et al. Loss of T-cell protein tyrosine phosphatase induces recycling of the platelet-derived growth factor (PDGF) β-receptor but not the PDGF α-receptor. Mol Cell Biol. 2006;17:4846–55. 10.1091/mbc.E06-04-0306 PMC1635401 16971512

[CR6] Li Z, Yang H, Li X, She T, Tao Z, Zhong Y, et al. Platelet-derived growth factor receptor β-targeted positron emission tomography imaging for the noninvasive monitoring of liver fibrosis. Eur J Nucl Med Mol Imaging. 2024;51(6):1530–43. 10.1007/s00259-023-06577-7. 38189910 10.1007/s00259-023-06577-7

[CR7] Lindborg M, Cortez E, Höidén-Guthenberg I, et al. Engineered high-affinity affibody molecules targeting platelet-derived growth factor receptor β in vivo. J Mol Biol. 2011;407(2):298–315. 10.1016/j.jmb.2011.01.033. 21277312 10.1016/j.jmb.2011.01.033

[CR8] Parola M, Pinzani M. Liver fibrosis: pathophysiology, pathogenetic targets and clinical issues. Mol Aspects Med. 2019;65:37–55. 10.1016/j.mam.2018.09.002. 30213667 10.1016/j.mam.2018.09.002

[CR9] Pinzani M, Milani S, Herbst H, DeFranco R, Grappone C, Gentilini A, et al. Expression of platelet-derived growth factor and its receptors in normal human liver and during active hepatic fibrogenesis. Am J Pathol. 1996;148(3):785–800. 8774134 PMC1861723

[CR10] Sanyal AJ, Shankar SS, Yates KP, Bolognese J, Daly E, Dehn CA, et al. Diagnostic performance of circulating biomarkers for non-alcoholic steatohepatitis. Nat Med. 2023;29(10):2656–64. 10.1038/s41591-023-02539-6. 37679433 10.1038/s41591-023-02539-6 PMC10579051

[CR11] Selvaraju RK, Bulenga TN, Espes D, Lubberink M, Sörensen J, Eriksson B, et al. Dosimetry of [68Ga]Ga-DO3A-VS-Cys40-Exendin-4 in rodents, pigs, non-human primates and human - repeated scanning in human is possible. Am J Nucl Med Mol Imaging. 2015;5:259–69. 26069859 PMC4446394

[CR12] Strand J, Varasteh Z, Eriksson O, Abrahmsen L, Orlova A, Tolmachev V. Gallium-68-labeled affibody molecule for PET imaging of PDGFRβ expression in vivo. Mol Pharm. 2014;11(11):3957–64. 10.1021/mp500284t. 24972112 10.1021/mp500284t

[CR13] Tolmachev V, Varasteh Z, Honarvar H, et al. Imaging of platelet-derived growth factor receptor β expression in glioblastoma xenografts using affibody molecule ^111^In-DOTA-Z09591. J Nucl Med. 2014;55(2):294–300. 10.2967/jnumed.113.121814. 24408895 10.2967/jnumed.113.121814

[CR14] Tsuchida T, Lee YA, Fujiwara N, Ybanez M, Allen B, Martins S, Fiel MI, Goossens N, Chou HI, Hoshida Y, Friedman SL. A simple diet- and chemical-induced murine NASH model with rapid progression of steatohepatitis, fibrosis and liver cancer. J Hepatol. 2018;69(2):385–395. 10.1016/j.jhep.2018.03.011. Epub 2018 Mar 21. Erratum in: J Hepatol. 2018;69(4):988. https://doi.org/10.1016/j.jhep.2018.07.010 10.1016/j.jhep.2018.03.011 PMC6054570 29572095

[CR15] Tsuchida T, Friedman SL. Mechanisms of hepatic stellate cell activation. Nat Rev Gastroenterol Hepatol. 2017;14:397–411. 28487545 10.1038/nrgastro.2017.38

[CR16] Wang S, Li K, Pickholz E, Dobie R, Matchett KP, Henderson NC, et al. An autocrine signaling circuit in hepatic stellate cells underlies advanced fibrosis in nonalcoholic steatohepatitis. Sci Transl Med. 2023;15(677):eadd3949. 10.1126/scitranslmed.add3949. 36599008 10.1126/scitranslmed.add3949 PMC10686705

[CR17] Wegrzyniak O, Rosestedt M, Eriksson O. Recent progress in the molecular imaging of nonalcoholic fatty liver disease. Int J Mol Sci. 2021;22(14):7348. 10.3390/ijms22147348. 34298967 10.3390/ijms22147348 PMC8306605

[CR18] Wegrzyniak O, Zhang B, Rokka J, et al. Imaging of fibrogenesis in the liver by [^18^F]TZ-Z09591, an Affibody molecule targeting platelet derived growth factor receptor β. EJNMMI Radiopharm Chem. 2023;8(1):23. 10.1186/s41181-023-00210-6. 37733133 10.1186/s41181-023-00210-6 PMC10513984

[CR19] Wegrzyniak O, Lechi F, Mitran B, et al. Non-invasive PET imaging of liver fibrogenesis using a RESCA-conjugated Affibody molecule. iScience. 2024;27(5):109688. 10.1016/j.isci.2024.109688. 38660405 10.1016/j.isci.2024.109688 PMC11039342

[CR20] Wong L, Yamasaki G, Johnson RJ, Friedman SL. Induction of beta-platelet-derived growth factor receptor in rat hepatic lipocytes during cellular activation in vivo and in culture. J Clin Invest. 1994;94(4):1563–9. 10.1172/JCI117497. 7929832 10.1172/JCI117497 PMC295310

[CR21] Xu L, Hui AY, Albanis E, Arthur MJ, O’Byrne SM, Blaner WS, et al. Human hepatic stellate cell lines, LX-1 and LX-2: new tools for analysis of hepatic fibrosis. Gut. 2005;54(1):142–51. 10.1136/gut.2004.042127. 15591520 10.1136/gut.2004.042127 PMC1774377

[CR22] Yashaswini CN, Qin T, Bhattacharya D, Amor C, Lowe S, Lujambio A, et al. Phenotypes and ontogeny of senescent hepatic stellate cells in metabolic dysfunction-associated steatohepatitis. J Hepatol. 2024;81(2):207–17. 10.1016/j.jhep.2024.03.014. 38508241 10.1016/j.jhep.2024.03.014 PMC11269047

