# Supplementary material for: PDGFRß targeted positron emission tomography as a non-invasive biomarker for activated hepatic stellate cells: lasts steps before clinical translation
Source: EJNMMI Radiopharm Chem. 2025 Dec 13;10:80. doi: 10.1186/s41181-025-00410-2 (PMC12711615; doi:10.1186/s41181-025-00410-2)
Supplement: Supplementary file 1 — Additional file 1 (DOCX 6400 KB) [file 41181_2025_410_MOESM1_ESM.docx]

**Supplementary Data**

**PDGFRß targeted Positron Emission Tomography as a non-invasive biomarker for activated Hepatic Stellate Cells – lasts steps before clinical translation**

Chittampalli N. Yashaswini et al.

**Supplementary Methods**

*Surface plasmon resonance studies on DOTA-Cys-ATH001*

The immobilization was made by standard procedures described by the vendor Cytiva AB using a T200 SPR instrument. All three target proteins, rhPDGFRβ (recombinant human), rhPDGFRα and rmPDGFRβ (recombinant murine) were diluted to 10 µg/ml in acidic immobilization buffer and immobilized with amine coupling on a CM5-sensorchip. For the human receptors immobilization buffer was 10 mM acetate pH 5.0 and for the murine receptor it was 10 mM Acetate pH 4.5. In order to reduce mass transport limitations under affinity experiments the immobilization density was kept to a low level of 400-600 RU. The instrumental buffer was HBS-EP+ (10 mM HEPES, 0.15 mM NaCl, 3 mM EDTA and 0.05% v/v Surfactant P20, pH 7.4). This buffer was maintained under both immobilization and affinity experiments.

When studying the affinity of DOTA-Cys-ATH001 binding to the proteins a method of multi-cycle kinetics with a broad concentration range of 3000 nM diluted in a series of 3 times to 0.15 nM, was used. This was sufficient to reach a saturation level. In the multi-cycle kinetics method each sample was injected over the receptor surface for 60 s and left to dissociate for 180 s with a flowrate of 30 µl/min. After injection the interaction between sample and target was broken with a 30 s long injection of 10 mM NaOH and 1 M NaCl. In order to let the surface stabilize the surface left in buffer flow for 480 s before start of a new sample cycle.

The competition between DOTA-Cys-ATH001 and PDGF-BB (the endogenous ligand) for binding to PDGFRβ was also examined using a SPR assay. rhPDGF-BB was immobilized on a CM5-chip. A concentration series of samples with 100 nM of the receptor rhPDGFRβ and DOTA-Cys-ATH001 at 11 concentrations between 1 and 1024 nM were injected over the ligand surface. Before and after the concentration series, 100 nM of the receptor was injected without DOTA-Cys-ATH001. The first of those was set to 100% and used to recalculate the signal of the other samples to percent relative this first sample. The second was used to evaluate the quality of the experiment.

A simplistic estimation of IC_50_ was made by removing the data where the DOTA-Cys-ATH001 concentrations was so high that it had already competed out the interaction between rhPDGF-BB and its receptor rhPDGFRb completely. The remaining data point were fitted to a polynomial equation from where the Z09591 concentration at 50% signal was calculated.

*Gallium-68 radiolabeling and shelf-life of DOTA-Cys-ATH001*

DOTA-Cys-ATH001 was radiolabeled by chelation of Gallium-68 into the DOTA moiety conjugated at the C-terminal. Gallium-68 for radiolabeling was generated by elution of Germanium-68/Gallium-68 generator (IGG100, WIIK Pharma, Denmark) using 0.1M metal-free HCl (prepared from Hydrochloric acid 32%, NORMATOM and Water, Ultra Trace Elemental Analysis Grade, Fisher Chemical).

A stock solution of 20-40µl DOTA-Cys-ATH001 precursor (7-14 nmol) was diluted with 200-250μl of 1 M sodium acetate buffer (pH 4.6, Honeywell Fluka). Next, 200-300µl of Gallium-68 eluate was added (100-200 MBq depending on generator age) and allowed to incubate for 15 minutes at 60-95°C. The radiolabeling efficiency was optimized with respect to labeling temperature.

The crude product was purified using a NAP-5 size-exclusion column (Cytiva). Quality control was conducted using Reversed-Phase High Performance Liquid Chromatography (RP-HPLC) and the radiochemical yield and molar activity of [^68^Ga]Ga-DOTA-Cys-ATH001 was calculated for each synthesis. [^68^Ga]Ga-DOTA-Cys-ATH001 was finally formulated in PBS (max 5% EtOH) for preclinical experiments.

Stability of [^68^Ga]Ga-DOTA-Cys-ATH001 in the formulation buffer at room temperature (RT) with and without EDTA challenge was monitored for 60 minutes

*In vitro plasma stability of [^68^Ga]Ga-DOTA-Cys-ATH001*

Plasma stability of [^68^Ga]Ga-DOTA-Cys-ATH001 was evaluated by incubation with human or rat plasma for up to 120 minutes. Human plasma (anonymized samples) was acquired from the local hospital blood bank. Rat plasma was acquired from healthy rats by heart puncture in anesthetized animals. The plasma was extracted from the blood by centrifugation (3000xg 2min, 4 °C), and the plasma either used fresh or frozen at -80°C for later use.

The detailed protocol for was described previously (1). Briefly, 400µl of plasma was added to 100 µl of [^68^Ga]Ga-DOTA-Cys-ATH001 formulation. The mixtures were incubated at 37°C for 60 or 120 minutes. After incubation, the mixture was added acetonitrile (CAN) at a ratio of 1:1 for precipitation of plasma proteins. The sample was centrifuged (16000 x g, 2 min, 4°C) and the supernatant filtered through a 2X0.2 µm nylon membrane filter. Five µl of the processed sample was used for HPLC analysis of purity.

*In vitro and in vivo plasma analysis using HPLC*

The stability study was performed by an HPLC (VWR HITACHI) system, equipped with a manual injection loop (max sample volume injected 20µl), a UV detector (wavelength range:210-280nm), a Chromaster pump 5110, and a radio-detector probe FC-3300 NaI/PMT using an Eckert /Ziegler dynamic range module for low (100k CPM) and High selected activities (12M CPM).

The sample analysis was based on a reverse phase column Vydac C4 214 MS Grace i.d. 5 mm (50 mm × 4.6 mm) using a gradient elution method. There were 2 mobile phases, Mobile phase A corresponding to 0.1% trifluoroacetic acid (TFA) in de-ionized water, and mobile phase B was 0.1% TFA in acetonitrile (MeCN). Elution was carried out at a flow rate 1ml/min, using the following elution program: 0-10 min (10%-90% B) and 10-12min (90%-10%B). Under these conditions, the retention times of free Gallium-68 and intact [^68^Ga]Ga-DOTA-Cys- ATH001were in the range of 0.6-0.8 min and 5.3-5.7 min respectively. The Agilent OpenLAB Chromaster EZChrome Edition software was used for acquisition and analysis.

*Culturing of U87 cells*

U87 cells (ATCC, USA), an adherent glioma cell line known to express PDGFRβ, were used to develop in vitro assays for [^68^Ga]Ga-DOTA-Cys-ATH001 validation. The U87 cells were used for several purposes. Firstly, cells were used for developing an in vitro cell binding assay. Secondly, U87 cells were frozen as pellets and processed using a microtome into 10µm sections on object glasses, to be used as positive controls sections in autoradiography binding studies. Thirdly, U87 cells were implanted in immune-incompetent mice to generate a xenografts model of PDGFRβ positive tumors. Details on the assays are given below.

U87 cells were cultured at 37°C and 5% CO₂ in Eagle’s minimum essential medium (ATCC, USA) supplemented by 10% fetal bovine serum (Sigma Aldrich), as well as 1 and 1% penicillin-Streptomycin (Biochrom, Berlin, Germany). Cells were trypsinized for detachment using Trypsin-EDTA (0.25%, Gibco, UK) during culturing and assays.

*In vitro cell binding studies*

A saturation binding study was performed with U87 cells to verify binding and affinity of [^68^Ga]Ga-DOTA-Cys-ATH001 to PDGFRβ. U87 cells were seeded at a density of 50000 cells / mL and cultured in 2 mL in 6-well plates and incubated at 37°C in a sterile cabinet with 5% CO₂. For the assay, 0.1 million cells were incubated with [^68^Ga]Ga-DOTA-Cys-ATH001 in culturing media, in different radioligand concentrations ranging from 100pM to 10nM. Identical wells were pretreated for 10 minutes with 16µM of unlabeled DOTA-Cys-ATH001 to induce complete blocking of all available PDGFRβ. All conditions were performed as triplicates. After incubating the U87 cells with the radioligand for 60 minutes at 37°C with 5% CO₂, the radioactive media was discarded, the cells washed with PBS, trypsinized, and collected for measurement in a gamma counter.

Cell binding of [^68^Ga]Ga-DOTA-Cys-ATH001 was quantified as fmol/ million cells using an internal standard of known radioactivity. The total and non-specific binding at each concentration of radioligand was expressed as a mean of the triplicates. The dissociation constant was estimated using the total and non-specific binding model in GraphPad.

*Biodistribution and dose finding study in mice*

In order to select the administered dose for future experiments, a dose finding study was performed in mice. From experience, murine spleen is immunopositive for PDGFRβ and can thus be used as a positive control for detecting PDGFRβ protein in vivo.

Mice (Balb/c, female, 25-30g, n=20) were administered 0.4±0.1 MBq of [^68^Ga]Ga-DOTA-Cys-ATH001, where the corresponding peptide mass was set or spiked to different amounts (0.2 µg, 0.3 µg, 1.4 µg, 2.6 µg, 200 µg, n=4 mice per group). After 60 minutes, the mice were euthanized, organs (blood, lungs, liver, spleen, kidneys and muscle) collected, weighed and measured by gamma counter and expressed as %ID/g.

*Histology and immunohistochemistry*

In several assays, selected tissues were further processed after PET or gamma counter measurement, to allow histology or immunohistochemical analysis. The tissue of interest was e.g. liver, or U87 tumors, while murine spleen often was added as internal PDGFRβ positive control, while muscle tissue was added as PDGFRβ negative control. The following general protocol was used: Tissues were fixed in 4% paraformaldehyde (PFA) for around 24 hours, then dehydrated in 70% ethanol, and embedded in paraffin. Paraffin blocks were processed into sections (4 μm) and stained with hematoxylin-eosin (H&E), Sirius Red (SIR), and/or Masson’s Trichrome (MT) at the Uppsala University Hospital pathology platform according to standard methods used for clinical pathology. Sections were immunostained for PDGFRβ using the DAKO Autostainer Link48 and Envision FLEX, high pH detection kit system (DAKO #K8000, Agilent). The sections were incubated with anti-PDGFRβ antibody (1:300 dilution for 60 minutes, ab32570, Abcam) followed by a horseradish peroxidase (HRP)-conjugated secondary antibody (Envision FLEX RTU). The sections were digitally imaged using the Nanozoomer S60 (Hamamatsu) at 20.0× magnification and visualized using QuPath-0.2.3 of Image J.

*Plasma concentrations and human predicted dosimetry based on rat and pig biodistribution data*

The plasma concentrations of DOTA-Cys-ATH001 in blood in rat and pig were estimated from delineation of the blood pool in the dynamic PET imaging scans described above. The plasma concentration of [^68^Ga]Ga-DOTA-Cys-ATH001 in kBq/mL was converted to ng/mL by the molar activity of the tracer at the time of injection (in MBq/nmol) in each animal.

The predicted human dosimetry of [^68^Ga]Ga-DOTA-Cys-ATH001 was interpolated from the biodistribution data from PET imaging of rat and pig above as described in detail previously (2). Briefly, the residence time in individual organs was calculated by estimation of the area under the curve of the dynamic non-decay corrected time-activity curves from each tissue, normalized for human organ volumes. The human dosimetry and effective dose of [^68^Ga]Ga-DOTA-Cys-ATH001 was estimated using (OLINDA/EXM).

*NOAEL and pharmacokinetics of DOTA-Cys-ATH001*

The PET tracer [^68^Ga]Ga-DOTA-Cys-ATH001 was intended to be injected intravenously at microdosing (<100µg, target dose of 1 µg/kg). The main constituent of the injected compound will be unchelated DOTA-Cys-ATH001, rather than DOTA-Cys-ATH001 complexed with radioactive Gallium-68. Therefore, a toxicology study, including PK/ toxicokinetic (TK) analyses, was performed according to the ICH M3 (R2) guidelines for microdosing regulatory requirements where unchelated DOTA-Cys-ATH001 was administered as an i.v. bolus to groups of Wistar Han rats (see Supplementary Table 1 for details).

*Pharmacokinetic analysis of DOTA-Cys-ATH001 in rats*

The toxicology study included animals for PK/ toxicokinetic (TK) analyses. Six male and six female rats were divided into groups of three/sex and administered a single iv bolus dose of 100 or 1000 µg/kg of DOTA-Cys-ATH001 (Supplementary Table 1). Blood samples for plasma analysis (K-EDTA tubes), were taken at 5, 15 and 30 minutes and at 1, 3, 6 and 24 hrs post dose.

DOTA-Cys-ATH001 in rat plasma samples was analyzed by LC-MS/MS after precipitation with ACN:MeOH (1:1) + 0.5 % formic acid solution. The calibration range of the method was defined from 200 to 10 000 ng/mL for DOTACys-ATH001. A total of 81 plasma samples were analysed for DOTA-Cys-ATH001 in two valid chromatographic runs. Each chromatographic run included a set of calibration standards, blanks (blank rat plasma), zero sample (blank rat plasma spiked with internal standard), quality control samples at four different concentrations levels (600, 1600, 4000, and 7500 ng/mL). The chromatographic runs were accepted according to the acceptance criteria defined for the calibration curve and quality control samples.

**Supplementary References**

1. Wegrzyniak O, Lechi F, Mitran B, et al. Non-invasive PET imaging of liver fibrogenesis using a RESCA-conjugated Affibody molecule. *iScience*. 2024;27(5):109688.
2. Selvaraju RK, Bulenga TN, Espes D, Lubberink M, Sörensen J, Eriksson B, Estrada S, Velikyan I, Eriksson O. Dosimetry of [68Ga]Ga-DO3A-VS-Cys40-Exendin-4 in rodents, pigs, non-human primates and human - repeated scanning in human is possible. Am J Nucl Med Mol Imaging. 2015;5:259-269

**Supplementary Figures**

**Supplementary Figure 1.** Concept of targeting PDGFRβ by PET for non-invasive assessment of active fibrosis in liver (A). Schematic structure of [^68^Ga]Ga-DOTA-Cys-ATH001 (B)

**Supplementary Figure 2.** Overview of the FAT-MASH model (A). Transcription of *Pdgfrb* is increased in HCC tumor and stroma in 24 week FAT-MASH liver, compared to in liver in control mice with a regular diet (B)

**Supplementary Figure 3.** Representative HPLC chromatogram and mass spectrometry analyses of DOTA-Cys-ATH001 precursor.

**Supplementary Figure 4.** snRNAseq analysis of transcription of *Pdgfb* and *Pdgfd* transcripts, encoding the endogenous ligands of PDGFRβ, in liver of FAT-MASH mice.

**Supplementary Figure 5.** snRNAseq analysis of *PDGFRβ* transcripts in human MASH liver. Clustering of human snRAseq data (A). *PDGFRβ* expression was mainly seen in the HCS cluster (B). Percent of cells in each cluster that expressed *PDGFRβ* (C). HSCs were further re-clustered into sub-populations: quiescent HSCs (qHSC healthy), quiescent precursors of activated HSCs (qHSC pre-act), activated HSCs (aHSCs), activated and deactivated HSCs (aHSCs + dHSCs) and senescent HSCs (sHSCs) (D). High *PDGFRβ* expression was solely found in aHSCs and aHSCs + dHSCs, while *PDGFRβ* expression was low in sHSCs and absent in all other HSCs including qHSCs (E-F). The clusters in panel A and D could be further divided into cells from MASH or healthy controls, which demonstrated that *PDGFRβ* expression was primarily found in HCSs in individuals with MASH (G), and in these subjects more specifically aHSCs and sHSCs (H).

**Supplementary Figure 6.** snRNAseq analysis of transcription of *PDGFB* and *PDGFD* transcripts, encoding the endogenous ligands of PDGFRβ, in human MASH liver.

**Supplementary Figure 7.** snRNAseq analysis of putative imaging biomarker targets for detection of active fibrosis, integrins α_v_β_3_ and α_v_β_6_, in liver of FAT-MASH mice.

**Supplementary Figure 8.** Representative sensorgrams showing the interaction between DOTA-Cys-ATH001 and recombinant human PDGFRβ (A), recombinant murine PDGFRβ (B) and recombinant human PDGFRα (C). Raw data sensorgrams for one of the two experiments depicted in 7E. The colours of the sensorgrams are blue when only rhPDGFRβ are in the sample and yellow when the sample contain both samples, DOTA-Cys-ATH001 and increasing concentrations of rhPDGFRβ (D). Reference and blank subtracted response of the interaction of immobilized rhPDGF-BB and a sample mixture of recombinant human PDGFRβ and the Affibody molecule DOTA-Cys-ATH001. The receptor concentration is 100 nM in every sample but the concentration of DOTA-Cys-ATH001 was varied from 0 to 1024 nM. Its concentration is marked beside every measurement point. Control samples with only the receptor injected over the ligand at the surface are shown at the top (E).

**Supplementary Figure 9.** Optimization of Gallium-68 radiolabeling of [^68^Ga]Ga-DOTA-Cys-ATH001 with respect to temperature (A), and representative HPLC chromatograms of product (B).

**Supplementary Figure 10.** Shelflife of [^68^Ga]Ga-DOTA-Cys-ATH001 in formulation, either alone or in the presence of EDTA (A). Representative HPLC chromatograms (B).

**Supplementary Figure 11.** In vitro stability of [^68^Ga]Ga-DOTA-Cys-ATH001 in human and rat plasma for up to two hours (A). Representative HPLC chromatograms (B).

**Supplementary Figure 12.** In vitro binding of [^68^Ga]Ga-DOTA-Cys-ATH001 to U87 cells at concentrations up to 10nM radioligand, either alone or co-incubated with Cys-ATH001 in excess to induce blocking.

**Supplementary Figure 13.** Representative autoradiograms of [^68^Ga]Ga-DOTA-Cys-ATH001 binding to liver, spleen and muscle sections from mice, either healthy (A), following 6 weeks of CCl_4_ (B) or after 2 weeks regression (CCl_4_ removal after 6 weeks treatment) (C). The degree of liver fibrosis was evaluated by SIR staining and PDGFRβ expression assessed by immunostaining.

**Supplementary Figure 14.** In vivo dose finding study in mice, using PDGFRβ positive spleen as a proxy for any PDGFRβ positive lesion. [^68^Ga]Ga-DOTA-Cys-ATH001 binding in relevant tissues were evaluated 60 minutes after injected by gamma counting post-mortem.

**Supplementary Figure 15.** Representative PET/MRI images of [^68^Ga]Ga-DOTA-Cys-ATH001 binding in U87 xenograft carrying immunodeficient mice, either tracer alone or after co-injection of 1mg/kg unlabeled Cys-ATH001 for self-blocking (A). Uptake of [^68^Ga]Ga-DOTA-Cys-ATH001 in relevant tissues quantified as %ID/g (B). Representative immunostaining demonstrating PDGFRβ protein expression in explanted U87 xenografts and mouse spleen (C).

**Supplementary Figure 16.** In vivo stability of [^68^Ga]Ga-DOTA-Cys-ATH001 in rat plasma for up to two hours after injection (A). Representative HPLC chromatograms (B).

**Supplementary Table 1.** Design of the microdosing acute toxicology study with DOTA-Cys-ATH001.

| Study type | Group number | Group | Route | Dose (µg/kg) | Time of euthanasia | Number of animals | |
| --- | --- | --- | --- | --- | --- | --- | --- |
|  |  |  |  |  |  | Males | Females |
| Main | 1 | Control | i.v. | - | 24 h | 10 | 10 |
|  | 2 | Low dose | i.v. | 10 | 24 h | 10 | 10 |
|  | 3 | Mid dose | i.v. | 100 | 24 h | 10 | 10 |
|  | 4 | High dose | i.v. | 1000 | 24 h | 10 | 10 |
| Recovery | 1 | Control recovery | i.v. | - | Day 14 | 5 | 5 |
|  | 4 | High dose recovery | i.v. | 1000 | Day 14 | 5 | 5 |
| TK | 5 | Mid dose | i.v. | 100 | N/A | 3 | 3 |
|  | 6 | High dose | i.v. | 1000 | N/A | 3 | 3 |
